# The impact of chronic obstructive pulmonary disease (COPD) and its associated lung hyperinflation on cardiac structure and function

**DOI:** 10.1186/1532-429X-17-S1-P353

**Published:** 2015-02-03

**Authors:** Ian S Stone, Mohammed Y Khanji, Wai-Yee James, Armida Balawon, Redha Boubertakh, Neil C Barnes, Steffen E Petersen

**Affiliations:** 1 NIHR CVBRU Centre For Advanced Cardiovascular Imaging, William Harvey Research Institute, London, UK; 2 Department of Respiratory Medicine, London Chest Hospital, London, UK; 3 Global Respiratory Franchise, GlaxoSmithKline, Stockley Park, UK

## Background

Significant cardiovascular morbidity and mortality exists in COPD, independent of traditional risk factors, although the mechanisms are poorly understood. Lung hyperinflation, the presence of supra-normal residual volumes (RVol) at the end of expiration, is one postulated mechanism. We sought to investigate the impact of COPD on cardiac structure and function compared to a control group with equivalent cardiovascular risk and look at the relative contributions of lung hyperinflation, defined as RVol per cent predicted value >120 %, as well as traditional spirometric measures.

## Methods

45 consecutive COPD patients with lung hyperinflation randomised to the DEFLATA study underwent cardiac magnetic resonance at 1.5 T, spirometric and body plethysmographic assessment at baseline. 9 were excluded from this analysis due to a history of known cardiovascular disease and/or atrial fibrillation and the remaining 36 were compared to 44 consecutive patients with increased cardiovascular risk and normal spirometry who underwent CMR as part of the HAPPY London Study.

## Results

There was no difference in age, gender, renal function or cardiovascular risk (QRisk) scores between the groups. Decreased Right ventricular (RV) and Left ventricular (LV) indexed end diastolic volumes (EDVI) were found in the COPD group (RVEDVI 77±18 vs 88±19 ml p=0.009; LVEDVI 63±14 vs 77±15 ml p<0.0001) with decreased but preserved LV systolic function (LV Ejection fraction (EF) 60±7 vs 64±7 % p=0.005). RVEF was increased (63±7 vs 55±7 ml P<0.0001) as a consequence of preserved stroke volume (RVSVI 48±12 vs 47±9 ml, p=0.71). In the COPD group a 10% increase in RVol per cent predicted resulted in a 1.97 ml, 1.66 ml and 1.36 ml reduction in RVEDVI, LVEDVI and Left Atrial (LA) ESVI respectively (RVEDVI β=-0.197, p=0.017; LVEDVI β=-0.166, p=0.018; LAESVI β=-0.136, p=0.008) independent of age, sex, oxygen saturations, and Qrisk score. Furthermore a relationship between Rvol and LAEF (β=-0.197, p=0.024) was found although not between LVEF or RVEF. Similar relationships were found for the expiratory flow limitation measure per cent-predicted Forced expiratory volume in 1 second (FEV1), although no relationship was found with regard to airflow obstruction (FEV1/Forced vital capacity).

## Conclusions

Morphological and functional differences exist between hyperinflated COPD patients and a control group with equivalent cardiac risk as defined by traditional risk factors. RVol and FEV1 per cent-predicted appear to be independently associated with cardiac chamber size. Effects are also seen on atrial function although right ventricular adaptation has ensured systolic function is preserved. Further studies are warranted to establish to what extent lung hyperinflation impacts on diastolic function and whether these changes are reversible.

## Funding

ISS has recieved a research grant from GSK. MYK is funded through Barts Charity.

